# Reduction in ACE2 expression in peripheral blood mononuclear cells during COVID-19 – implications for post COVID-19 conditions

**DOI:** 10.1186/s12879-024-09321-0

**Published:** 2024-07-03

**Authors:** Gulrayz Ahmed, Yasir Abdelgadir, Amro Abdelghani, Pippa Simpson, Jody Barbeau, Donald Basel, Christy S. Barrios, Brandon A Smith, Kala F Schilter, Rupa Udani, Honey V. Reddi, Rodney E. Willoughby

**Affiliations:** 1 Medical College of Wisconsin, Milwaukee, Wisconsin USA; 2 Pediatric Infectious Diseases, C450, Medical College of Wisconsin, PO Box 1997, Milwaukee, WI 53201-1997 USA

**Keywords:** COVID-19, Angiotensin-converting enzyme 2; Transcription, Genetic, Leukocytes, Mononuclear; Biomarkers, Post-acute COVID-19 syndrome, Matrix metalloprotease-9, Tissue inhibitor of metalloprotease-1, N-terminal peptide of procollagen III

## Abstract

**Background:**

Severe COVID-19 is uncommon, restricted to 19% of the total population. In response to the first virus wave (alpha variant of SARS-CoV-2), we investigated whether a biomarker indicated severity of disease and, in particular, if variable expression of angiotensin converting enzyme 2 (ACE2) in blood might clarify this difference in risk and of post COVID -19 conditions (PCC).

**Methods:**

The IRB-approved study compared patients hospitalized with severe COVID-19 to healthy controls. Severe infection was defined requiring oxygen or increased oxygen need from baseline at admission with positive COVID-19 PCR. A single blood sample was obtained from patients within a day of admission. ACE2 RNA expression in blood cells was measured by an RT-PCR assay. Plasma ACE1 and ACE2 enzyme activities were quantified by fluorescent peptides. Plasma TIMP-1, PIIINP and MMP-9 antigens were quantified by ELISA. Data were entered into REDCap and analyzed using STATA v 14 and GraphPad Prism v 10.

**Results:**

Forty-eight patients and 72 healthy controls were recruited during the pandemic. ACE2 RNA expression in peripheral blood mononuclear cells (PBMC) was rarely detected acutely during severe COVID-19 but common in controls (OR for undetected ACE2: 12.4 [95% CI: 2.62-76.1]). ACE2 RNA expression in PBMC did not determine plasma ACE1 and ACE2 activity, suggesting alternative cell-signaling pathways. Markers of fibrosis (TIMP-1 and PIIINP) and vasculopathy (MMP-9) were additionally elevated. ACE2 RNA expression during severe COVID-19 often responded within hours to convalescent plasma. Analogous to oncogenesis, we speculate that potent, persistent, cryptic processes following COVID-19 (the renin-angiotensin system (RAS), fibrosis and vasculopathy) initiate or promote post-COVID-19 conditions (PCC) in susceptible individuals.

**Conclusions:**

This work elucidates biological and temporal plausibility for ACE2, TIMP1, PIIINP and MMP-9 in the pathogenesis of PCC. Intersection of these independent systems is uncommon and may in part explain the rarity of PCC.

## Background

Initial COVID-19 outbreaks caused severe disease in only 19% of the population despite absence of prior immunity to SARS-CoV-2 and abruptly ceased spreading despite most of the population remaining susceptible [1]. Limited spread was described in COVID-19 outbreaks in closed facilities such as ocean ships and skilled nursing facilities [2] and intra-family transmission enhanced risk for disease, supporting a shared genetic or environmental susceptibility to severe disease [3]. The influence of non-communicable comorbid diseases and age to disease severity further underlines complex genetic, developmental, and environmental determinants of COVID-19 severity and its later effects. Cessation of outbreaks has been ascribed to physical distancing and masking. However, these interventions limit, but do not stop a highly communicable disease circulating in a universally susceptible population [1, 4]. The initial wave of local epidemics declined when seroprevalence to SARS2 approximated 20%, far below that needed for protection by herd immunity.

When the apparent spread of a rapidly communicable infectious disease such as COVID-19 stops abruptly, an alternative explanation to herd immunity is reduced genetic or developmental susceptibility to the disease among the remaining, unaffected population. This reduction might encompass relative resistance to infection by the virus or possibly, for respiratory viruses, resistance to severe disease among those infected. In aggregate, the science suggests universal susceptibility to infection by SARS-CoV-2 (true for all respiratory viruses) and points to differences in host response determining severity of disease [5].

We focused on angiotensin-converting enzyme 2 (ACE2), the principal cellular receptor for SARS2 [6]. ACE2 is expressed ubiquitously, but pathogenesis of acute COVID-19 primarily localizes to type 2 alveolar cells in the lungs expressing ACE2. Type 2 alveolar cells synthesize pulmonary surfactant and further differentiate into type 1 alveolar cells that constitute the interface for gas exchange. An early report found increases in ACE2 expression in the lung associated with numerous chronic comorbidities and as a function of increasing age, consistent with risk for severe COVID-19 [7]. We postulated that increased ACE2 expression, as the primary virus receptor, explained increased severity of COVID-19 for these patient demographics. Because chronic comorbidities also increase ACE2 expression, we further hypothesized that ACE2 expression induced by comorbidities with complex inheritance and environmental exposures would modify the genetic risk for severe disease. Intending to develop point-of-care testing for triage of the SARS2-infected population, we hypothesized that ACE2 RNA expression in blood would correlate with ACE2 RNA expression in lung type 2 alveolar cells, providing a mechanistically related biomarker for screening and triage.

When we applied our assay to blood from hospitalized subjects and volunteers, we instead measured reduced ACE2 expression in PBMC from subjects first hospitalized for COVID-19. Indeed, ACE2 expression rapidly increased with the use of convalescent plasma (CP), used widely in the early stages of the pandemic.

Post-COVID-19 conditions (PCC) are highly variable. These include objective syndromes such as pulmonary and cardiac fibrosis and delayed inflammatory syndromes such as MIS-C and MIS-A [8–11]. The majority of neurocognitive, pain and fatigue complaints comprising PCC do not correlate with objective functional tests, imaging, or routine inflammatory markers [9, 12, 13]. Pulmonary fibrosis, including that following COVID-19, is associated with perturbations of matrix metalloproteases (MMP) in tissues and blood. High concentrations of MMP and pulmonary fibrosis are both associated severe acute COVID-19, so the contribution of MMP to PCC is confounded [9]. Kawasaki disease (KD) resembles MIS-C and is also associated with dysregulated MMP, but most childhood COVID-19 is asymptomatic or mild. The mild antecedent SARS-CoV-2 infection minimizes confounding of the association of elevated MMP with PCC [8, 14].

Following reports of MIS-C and PCC, we hypothesized that the cryptic pro-inflammatory and pro-fibrotic tone from reduced ACE2 expression in blood cells might intersect with additional plasma biomarkers of equally cryptic vasculopathy and fibrosis to further clarify individual risks of PCCs in the population., We selected TIMP-1, PIIINP and MMP-9 for assay from among MMP biomarkers reported as abnormal during acute COVID. PIIINP was of particular interest given its apparent constitutive deficiency in Kawasaki survivors [15, 16].

## Methods

The study was approved at the Medical College of Wisconsin Institutional Review Board (protocol 38127). Cases were 48 adults aged 54-72 years. Inclusion criteria included patients > 18 years hospitalized for an oxygen requirement, or increased oxygen requirement from baseline, with first admission for COVID-19 confirmed by a nucleic acid amplification test (NAAT) for SARS-CoV-2 (WHO scores 4-7) [17]. Exclusion criteria for patients were hospitalizations for other indications (e.g., trauma) who tested positive for the virus by routine admission screening, prior hospitalization for COVID-19, and having SARS2 IgG detected early upon admission. Controls were healthy volunteers aged 35-50 years from the Department of Pediatrics at Medical College of Wisconsin. A broadcast email requested voluntary participation for anonymous blood sample donation and a brief health questionnaire of salient risk factors for COVID-19 [7].

### ACE2 PCR

RNA was extracted from whole blood via Maxwell Simply RNA blood Kit (Promega), and reverse transcribed following the iScript Advanced cDNA Synthesis kit (Bio-Rad). 2µL of cDNA was utilized in qPCR reactions performed in multiplex with 20x ACE2 primer/probe (IDT Hs.PT.58.27645939) and 5x HPRT1 primer/probe (IDT Hs.PT.58v.45621572) as an internal control. Conditions consisted of initial denaturation at 95°C for 30 seconds followed by 40 cycles of 95°C for 10 seconds, and 60°C for 60 seconds with fluorescence acquisition. All samples were run in duplicate and subjected to ΔΔCT calculation for relative fold expression of ACE2 gene target versus ACE2-expressing cell line HepG2 (Sigma Aldrich 85011430-DNA-5UG). HepG2 was selected as a commonly available cell line known to express ACE2 but not ACE1 [18, 19].

### ACE1 and ACE2 enzyme activities

Plasma was assayed in duplicate for ACE1 enzyme activity with or without the specific ACE1 inhibitor 10 mM captopril, using the fluorescent substrate Mca-RPPGFSAFK(Dnp)-OH [20] Plasma was assayed for ACE2 enzyme activity with 10 mM captopril, with or without the specific inhibitor DX600, using the fluorescent substrate Mca-YVADAPK(Dnp)-OH [21].

### TIMP-1, PIIINP and MMP-2 antigens

TIMP-1 was assayed using the Quantikine human TIMP-1 ELISA (R&D Systems, Minneapolis, MN) according to manufacturer instructions using heparin plasma diluted 1:100. PIIINP was assayed using the human procollagen type III N-terminal peptide ELISA (Novus Biologicals, Centennial, CO) according to manufacturer instructions using plasma diluted 1:10. Human 92 (pro-) and 82 kDa (active) MMP-9 proteins were assayed using the Quantikine human MMP-9 ELISA (R&D Systems, Minneapolis, MN) according to manufacturer instructions using plasma diluted 1:40. All samples were in quantifiable range.

### Data collection and statistics

Data were abstracted from the electronic medical record onto a standardized form and entered into a REDCap database, which was then scrubbed and analyzed using Stata SE 14 (College Station, TX). Research data were analyzed under a masking code, keyed into Excel database, and then linked within Stata using a code key. Missing data were not imputed. Categorical data were analyzed by Pearson Chi-square or Fisher exact test and most continuous data were not normally distributed, so analyzed by a Mann Whitney non-parametric test. Correlations and pairwise comparisons were measured by the Pearson correlation coefficient.

## Results

COVID-19 was detected in Wisconsin on March 1, 2020 (week 10). Healthy volunteers were recruited anonymously after expedited IRB review in week 20. The prospective cohort study opened on August 20, 2020 (week 30). Recruitment of subjects in the medical intensive care unit (ICU) was not allowed. We interrupted accrual on November 3, 2020 (week 45) for a pre-defined statistical review of the first 50 hospitalized subjects with severe COVID-19. A total of 56 subjects were recruited but 8 were excluded for second admission (*n*=1) or positive COVID-19 IgG (*n*=7). Demographics of the study population are included in Table 1. The study period aligned with infections by the alpha variant of SARS-CoV-2.

**Table 1:** Cohort demographics

	1. Control (*n*=72)	2. Hospitalized (*n*=48)	2a. Hospitalized, <70 (*n*=32)	* *P*<0.05 ** *P*<0.01 *** *P*<0.001 ns (not significant)
Age in years (median, IQR, min/max)	40 (35-50) 22/69	64 (54-72) 31/93	57.5 (50-64) 31/69	1 vs 2** 1 vs 2a***
Sex (% female)	72.2	60.4	56.2	1 vs 2 ns 1 vs 2a ns
Race (n, %)				1 vs 2*** 1 vs 2a***
White (MKE county: 64.2)	60 (83.3)	28 (58.3)	18 (56.2)	
Non-white (35.8)	6 (8.3)	20 (**41.7**)	14 **(43.8**)	1 vs 2*** 1 vs 2a***
Black (27.2)	4 (5.6)	13 (**27.1**)	8 (**25.0**)	
American Indian/ Native Alaskan (1.0)	0	1 (2.1)	1 (3.1)	
Asian (4.7)	2 (2.8)	**4 (8.3**)	**4 (12.5)**	
Other (2.9)	0	2 (4.2)	1 (3.1)	
Unknown	6 (8.3)	0	0	
Ethnicity				
Hispanic or Latino (MKE county 15.6)	0	**3 (6.2)**	**2 (6.2)**	
Not*	3	45 (93.8)	30 (93.8)	
Unknown	69	0	0	
Socioeconomic quintile (n, %)				
High 1		6 (12.5)	3 (9.4)	
2		9 (18.8)	5 (15.6)	
3		12 (13.0)	6 (18.8)	
4		10 (**20.3**)	7 (**21.9**)	
Low 5		17 (**35.4**)	11 (34.4)	
Comorbidities				
Body Mass Index (median (IQR) min/max		34.2 (28.7 -39.62) 21.6/53.2	34.8 (29.5-38.9) 21.6/48.4	
Obesity (BMI >30, n, %)		30 (66.7)	23 (71.9)	
Needs mobility support (%)		20.8	**3.1**	<70 vs >=70***
Home oxygen (%)		6.2	3.1	
Smoker (%)		2.0	3.1	
COPD (%)		12.5	6.2	
Asthma (%)	2.8	22.9	**18.8**	1 vs 2*** 1 vs 2a**
Diabetes (%)	2.8	37.5	**34.4**	1 vs 2*** 1 vs 2a***
Thyroid supplement (%)	5.6	18.8	**25.0**	1 vs 2* 1 vs 2a**
Hypertension (%)	15.5	64.6	**50.0**	1 vs 2*** 1 vs 2a***
Coronary artery disease (%)	1.4	22.9	12.5	1 vs 2*** 1 vs 2a* <70 y vs >=70 y***
Home anticoagulant (%)		12.5	9.4	
ACE inhibitor (n, %)		7 (14.6)	4 (12.5)	
Lisinopril dose (median, IQR, min/max)		20 (10-40) 5/40	5, (10, 20), 5/20	
Angiotensin receptor blocker (n, %)		7 (14.6)	4 (12.5)	
Losartan dose (*n*=6; median, min/max)		100, 25/100	100, 50/100	
Irbesartan (*n*=1) dose		300	na	
Cancer				
Prior (n, %)		3 (6.2)	2 (6.2)	
Remission (n, %)		4 (8.3)	3 (9.4)	
Under treatment (n, %)		6 (12.5)	5 (15.6)	
None		35 (72.3)	22 (68.8)	
Home immunosuppression (n, %)		7 (14.6)	6 (18.8)	
Home oral corticosteroids (n, %)		11 (22.9)	6 (18.8)	
Home inhaled corticosteroids (n, %)		8 (16.7)	4 (12.5)	
Close COVID contact:				
Yes (%)		33.3	31.2	
No (%)		31.3	40.6	
Unk (%)		35.4	28.1	
Blood Sampling				
Blood product transfused before sample (%)		56.2	50.0	
Time, COVID-19 testing to hospitalization (days; median, IQR, min/max)		2 (0-6) 0/16	3 (0-7) 0/16	
Time, hospitalization to sample (days; median, IQR, min/max)		2 (2-4) 0/36	2 (2-3) 0/19	
Severity of illness				
Increased oxygen requirement (%)		100	100	
Quick SOFA score (median, IQR, min/max)		1 (0-1) 0/2	1 (0-1) 0/2	
RR>22 on admission (%)		70.8	62.5	
Systolic BP < 100 (%)		8.3	6.25	
Altered mental status (%)		12.5	**3.1**	<70 y vs >=70 y**
Acute kidney injury (%)		25.0	21.9	
Renal replacement therapy (n, %)		1 (2.1)	0	
Treatment				
Corticosteroids (%)		89.6	87.5	
Convalescent plasma (%)		70.8	71.9	
Remdesivir (%)		37.5	46.9	
Zanubritinib (%)		10.4	15.6	
Tocilizumab (%)		2.1	0	

* *P*<0.05; ** *P*<0.01; *** *P*<0.001; ns (not significant)

**Fig. 1:**
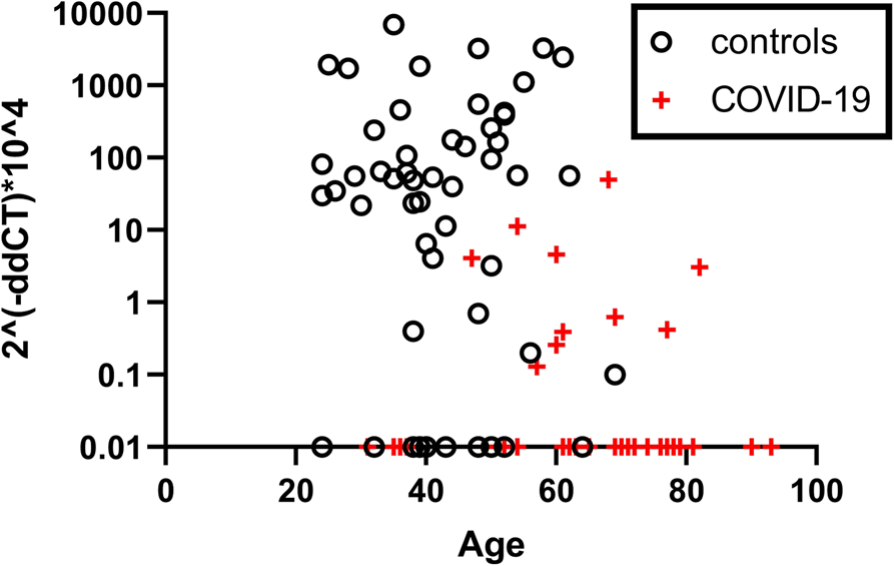
ACE2 gene expression in peripheral blood mononuclear cells from hospitalized subjects symptomatic with COVID-19 infection (*n*=48) or controls (*n*=72), by age

ACE2 RNA expression was detected in blood cells in 77% of the volunteers but only 28% of hospitalized adults with COVID-19 (Fig. 1, Table 2). When detected, ACE2 RNA levels were significantly lower in hospitalized subjects compared to healthy controls (Table 2). ACE2 RNA expression in blood of hospitalized subjects was rarely detected over age 70 years, the population with highest mortality for COVID-19. Given age disparities between study groups, we conducted a secondary analysis restricted to subjects aged less than 70 years (Tables 1&2) and the findings persisted. Circulating plasma ACE1 and ACE2 enzyme activities did not correlate with blood cell ACE2 RNA expression (Table 2).

**Table 2:** Subject outcomes and biomarkers

	1. Control (*n*=72)	2. Hospitalized (*n*=48)	2a. Hospitalized, <70 y (*n*=32)	*P<0.05 ** P<0.01 *** P<0.001 ns (not significant)
Outcome				
ICU (n, %)		9 (18.8)	3 (9.4)	<70 y vs >=70 y *
Time, hospitalization to ICU (days; median, IQR, min/max)		1 (0-1) 0/4	0, (0-0), 0/4	
Intubated among ICU patients (%)		2 (22.2)	0	
ECMO (%)		0	0	
Length of stay, alive (days, IQR, min/max)		6 (4-9) 2/47	5 (3-7) 2/47	
Deceased (n, %)		7 (14.6)	1 (3.1)	<70 y vs >=70 y ***
Length of stay, deceased (days)		9 (7-19) 6/39	6	
Renin Angiotensin System				
ACE 2 expression detected (n/N, %)	44/57 (77.2)	10/36 (27.8)	8/22 (36.4)	OR 1 vs 2 : 8.8 (95% CI 3.08-25.7) *** OR 1 vs 2a : 6.05 (95% CI 1.86-20.1) **
ACE2 expression detected, before plasma (n/N, %)		3/14 (21.4)	2/9 (22.2)	OR 1 vs 2 : 12.4 (95% CI : 2.62-76.1) ** OR 1 vs 2a : 11.8 (95% CI : 1.85-124) **
ACE2 expression detected, after plasma (n/N, %)		7/22 (31.8)	6/14 (42.8)	OR 1 vs 2 7.25 (95% CI : 2.16-25.2) ** OR 1 vs 2a 4.51 (95% CI : 1.11-18.5) *
ACE2 2^(-ddCT)*10k (median, IQR, min/max)	63.5 (24.2-406.3) 0.1/6925.5	1.86 (0.39-4.62) 0.13/50.0	0 (0-0.39) 0/50.0	1 vs 2: *** 1 vs 2a: ***
ACE1 enzyme nmol/h*ml (n, median, IQR, min/max)		*n*=45 11.08 (4.09-18.7) 0/25.1	*N*=33 10.47 (7.37-13.9) 0/22	
ACE2 enzyme nmol/h*ml (n, median, IQR, min/max)		*n*=46 2.99 (1.70-3.93) 0/9.76	*n*=34 3.28 (1.76-4.00) 0/9.76	
Vasculitis/ Fibrosis Systems				
MMP-9 ng/ml (n, median, IQR, min/max)		*n*=42 153.0 (50.8-191.5) 12.2/840	*n*=30 137.8 (46.8-166.2) 12.2/840	
TIMP-1 ng/ml (n, median, IQR, min/max)		n=42 444 (323-535) 194/852	*n*=30 437 (323-524) 194/852	
PIIINP ng/ml (n, median, IQR, min/max)		*n*=42 4.75 (4.11-5.21) 2.28/10.11	*n*=30 4.88 (3.93-5.36) 3.08/10.11	

* *P*<0.05; ** *P*<0.01; *** *P*<0.001

**Fig. 2:**
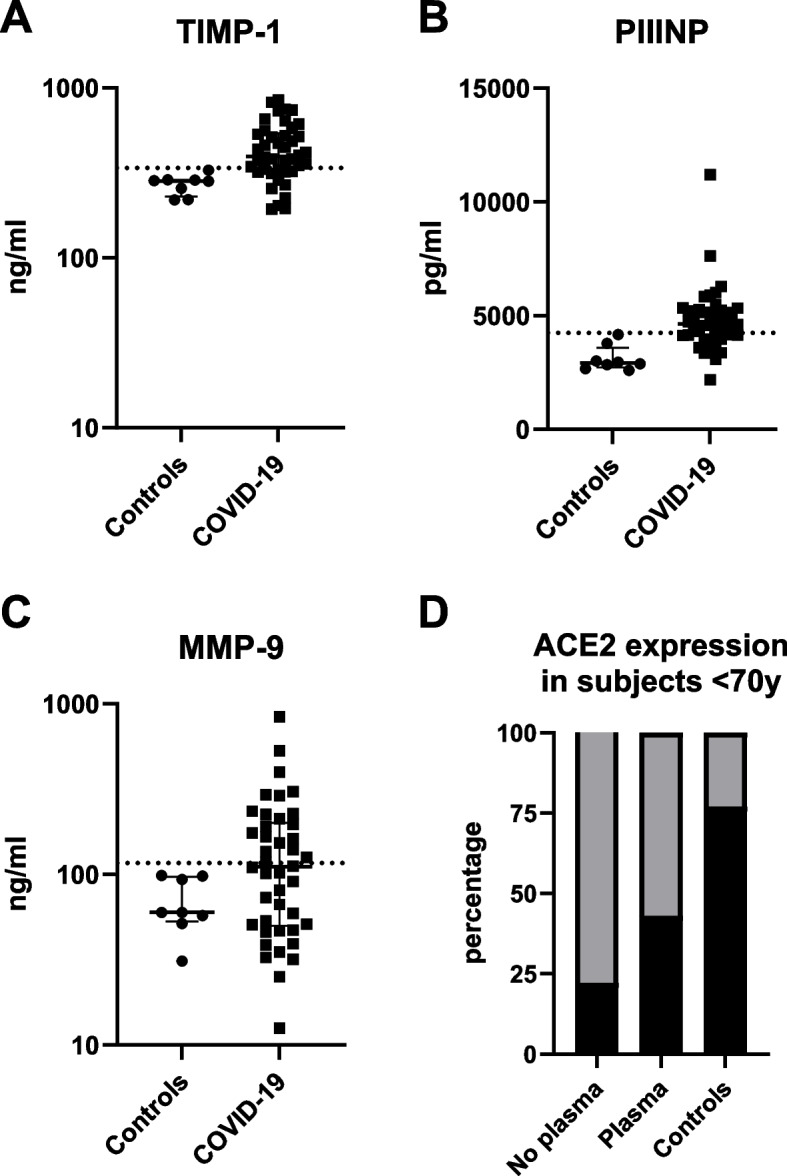
Plasma levels of, TIMP-1 (panel A) PIIINP (panel B) and MMP-9 (panel C) (*n*=42) and laboratory controls (*n*=8). Proportion of subjects aged less than 70 years with ACE2 RNA detected in blood cells (black), by early use of convalescent plasma, and healthy controls (panel D) (*n*=72)

ACE2 RNA expression in blood was tested shortly after hospitalization to avoid any perceived impact by investigational medical therapies (Table 1). Hospitalized subjects during the first COVID-19 wave were primarily treated with corticosteroids (89%) and convalescent plasma (70%). We did not detect effects from home corticosteroid use [22]. Results did not vary by treatments other than CP. Results did not vary by sample collection time, but roughly half of those hospitalized with severe COVID-19 received CP before our admission blood sample was drawn. Receipt of CP increased detectable ACE2 RNA expression from 21% to 32% of subjects within hours of administration (*p*=0.70). Examining the subset of subjects who had not yet received CP and under 70 years of age (i.e., capable of expressing ACE2 RNA in blood cells) resulted in a more extreme differential prevalence of ACE2 RNA expression: 77% of working volunteers versus 21% of those hospitalized with severe COVID-19. The odds ratio for undetected ACE2 RNA expression in those aged under 70 years with severe COVID-19 relative to controls was 11.8 (95% CI: 1.85-124) (Fig. 2; Table 2). ACE2 RNA expression after CP among those under 70 years increased from 22% to 43% (*p*=0.39).

Plasma TIMP-1 concentrations were higher in subjects with severe COVID-19 than controls (*p*=0.0032; Fig. 2). PIIINP was also elevated relative to controls (*p*=0.0017). Plasma levels of MMP-9 exceeded the normal range in 47% of hospitalized patients (*p*= .062). To synergize, these pathways and ACE2 expression should be independent. ACE2 RNA expression, MMP-9, TIMP-1 and PIIINP showed weak or no correlations in those hospitalized with severe COVID19 (Table 3).

**Table 3:** Correlations of ACE2 RNA detection and plasma biomarkers

Biomarker	ACE2	MMP-9	TIMP1	PIIINP
ACE2	1.0			
MMP-9	-0.325	1.0		
TIMP1	0.149	0.168	1.0	
PIIINP	.183	0.312*	0.210	1.0

Pairwise correlation of ACE2 RNA (detected), high MMP-9 (>117 pg/ml), high TIMP1 (>338 pg/ml), high PIIINP (>4241 pg/ml). Cutoffs exceed normal values. Significance *P<0.05

## Discussion

ACE2 RNA expression was only detected in blood cells in 21% of untreated adults hospitalized with severe COVID-19 and had lower expression than healthy controls when it was detected. This indicates a global shift to lower ACE2 RNA expression in blood cells of people severely ill with SARS-CoV-2 infection. ACE2 RNA expression in blood was rarely detected over age 70 years, the population with highest mortality for COVID-19. We did not anticipate undetectable ACE2 expression in blood given that ACE2 gene expression was known to increase with age and chronic comorbidities in most tissues, including the lung [23]. Local ACE2 RNA deficiency in blood cells suggested a modulatory or signaling role for ACE2 specific to blood cells. ACE2 RNA deficiency was associated with very high odds for severe COVID-19 in those less than 70 years (OR 11.8; 95% CI: 1.85-124) supporting its potential use as a point-of-care test for severe COVID-19.

Osman et al. also reported low ACE2 RNA expression in whole blood, PBMCs and monocytes during SARS-CoV-2 infection [24]. This group confirmed reduced monocyte ACE2 protein as well as RNA expression by flow cytometry, which we could not do because of biosafety regulations for COVID-19 at our institution at the time [24]. Taken together, the Marseilles and Milwaukee studies confirm that low ACE2 RNA expression in blood monocytes occurs early during COVID-19 and persists into the second week of illness. ACE2 counterbalances ACE1 effects within the RAS, being anti-inflammatory, anti-fibrotic, and antioxidant [6, 25]. Persistently reduced ACE2 expression in monocytes over weeks is conducive to unopposed inflammatory tone in the vasculature and has implications for PCC. Repeated SARS2 infections increased the risk of stroke and myocardial infarctions, consistent with our hypothesized cumulative, unopposed RAS inflammatory tone [26].

The mechanism of ACE2 expression in blood cells is discrete from classical peptide signaling within RAS. Circulating plasma ACE2 enzyme activity (regulating angiotensin II and angiotensin 1-7 peptides) did not correlate with blood cell RNA expression in this study. Plasma ACE2 activity likely originated from ecto-enzymes shed remotely from other tissues [27]. Blood cell expression of ACE2 may signal independently of angiotensin II, angiotensin 1-7 and other RAS peptides, involving cell-cell adhesion to endothelial cells of the vasculature [6, 25].

Monocyte dysregulation is believed to be central to the pathogenesis of severe COVID-19, so undetectable ACE2 RNA expression in blood cells during severe COVID-19 may be mechanistically important [28]. In model systems, low ACE2 RNA expression in transfected human monocytes increased ICAM-1, VCAM and M-CSF expression, monocyte adherence, and trans-endothelial migration [29]. The soluble ecto-enzyme, sACE2, binds integrin B1 adhesion molecules to modulate cell-endothelial interactions [30]; we presume the membrane bound form on monocytes does similarly. The ACE2 receptor includes a cytoplasmic collectrin-like domain that binds calmodulin for intracellular signaling. This indicates the potential for bi-directional signaling [25]. During COVID-19, intermediate and non-classical monocyte populations (expressing less ACE2 than classical monocytes) were decreased and correlated with increased disease severity [31, 32]. The reported changes in monocyte subpopulations favor higher expression of ACE2 and thus cannot explain the low ACE2 expression in blood cells we measured.

ACE2 RNA deficiency in blood cells may be constitutive in a subset of the population, possibly indicating genetic susceptibility to severe COVID-19 and/or to PCC. Among volunteers under age 70 (and at work), 23% did not have detectable ACE2 RNA in blood. This proportion of a healthy population approximates the 19% of immunologically naïve persons who developed severe COVID-19 after infection [1, 2, 47–49]. The prevalence of ACE2 RNA deficiency in blood in other healthy populations remains to be determined. Single cell RNAseq studies often could not detect ACE2 in blood cells [5, 50–53]. Studies using other methods (gene chip, RT-PCR assays, and flow cytometry) confirmed ACE2 RNA or protein expression in blood cells [29, 32, 54, 55]. Poor ACE2 detection by scRNAseq likely relates to low abundance mRNA transcripts (which we could not detect in 23% of healthy controls) and processing pipelines that limit noise inherent to high data/low N datasets [56].

If 19-23% of the population has constitutive ACE2 RNA deficiency predisposing to severe COVID-19, then that indicates that the remaining 54-58% of adults hospitalized with severe COVID-19 had blood ACE2 RNA suppressed by SARS-CoV-2 infection. This suppression was reversible. Convalescent plasma (CP) was associated with an increase in detectable ACE2 RNA expression from 22% to 43% of subjects aged under 70 years within hours of CP administration. This effect of CP on blood cell RNA expression may be underestimated by the early sampling time, supported by changes in lymphocytes which became evident 14-28 days after CP infusion [57]. There are many mechanisms postulated for CP effects, including antibodies to the SARS-CoV-2 virus, anti-idiotypic and autoimmune antibodies, antibodies modulating complement and phagocytosis, convalescent cytokines, chemokines, and growth factors, complement and clotting factors, and peptide and lipid hormones [58]. CP showed no efficacy against mortality during acute COVID-19, so direct antiviral effects as a mechanism for rapid changes in ACE2 expression by PBMC are unlikely [59]. CP resulted in lower IL-6, CXCL10, CCL2, IFN-gamma and M-CSF within 3-7 days [57, 61, 62]. Plasma concentrations of MMP-9, TIMP-1 and PIIINP were not affected by CP in this study, but our study design does not exclude delayed effects of CP over several days [57].

IVIG is a fractionated subset of healthy donor plasma enriched in anti-infective, anti-idiotypic, and complement-regulatory antibodies. IVIG modulates Fc-expressing immune cells and increases regulatory T-cells [63]. IVIG is highly effective in treating the pediatric PCC, MIS-C, with similar effects on KD [8, 60]. IVIG increased plasma sCD163, a monocyte/macrophage marker, in COVID-19 subjects; the same effect was also noted after IVIG treatment of Kawasaki disease [59, 64]. We are unaware of any report of the effect of CP or IVIG on the incidence of PCC.

The pro-fibrotic tone conferred by ACE2 deficiency in monocytes led us to consider additional pro-fibrotic pathways. Monocytes and macrophages are central to COVID pathogenesis, lung and cardiac fibrosis following COVID, express ACE and ACE2, and elaborate matrix metalloproteases [9, 10, 24, 28, 32, 65]. Kawasaki disease, a cognate for MIS-C, is characterized by altered MMP regulation, including what appears to be chronically elevated PIIINP [14–16, 35–39]. We investigated the co-incidence of markers intersecting COVID-19 and KD or MIS-C. All markers we studied persist over weeks, consistent with the delayed onset of PCC. We considered the progression to PCC to be analogous to theories of *initiation* and *promotion* during oncogenesis [46]. We speculate that SARS-CoV-2 might *initiate* disease processes in the context of age, sex, and pre-existing comorbidities that are then *promoted* by dysregulations in RAS, fibrosis or vasculopathy pathways. Tissue inhibitor of metalloproteases 1 (TIMP-1) and procollagen III amino-peptide (PIIINP) are markers of fibrosis, notably contributing to the enhanced liver fibrosis score [33]. TIMP-1 is also an independent cytokine [66]. PIIINP is well-studied in hypertensive cardiomyopathy, dilated cardiomyopathy, and congestive heart failure [34]. We found plasma TIMP-1 and PIIINP to be significantly elevated relative to controls, an observation previously reported [40–42, 67]. Plasma MMP-9 trended higher during COVID-19 in this study but reached statistical significance in other reports [40, 43–45, 67–70]. MMP-9 remained elevated at 3 months in studies published while this manuscript was under review [67–69]. Unlike other studies, these four markers for various pathways (monocyte ACE2, TIMP-1, PIIINP and MMP-9) were analyzed simultaneously. These markers were not well correlated, suggesting orthogonal pathways that might genetically intersect to cause PCC. In this study, 19% of subjects were coincidently abnormal using low stringency cutoffs for blood cell ACE2 expression, TIMP-1, PIIINP and MMP-9, a 5-fold enrichment for theoretical risk of PCC among those hospitalized for COVID19. Other promoters, second triggers, or more extreme biomarker thresholds are required to fully explain the rarity of PCC. Two groups reported prolonged dysfunction of the complement systems that were associated with PCC [71, 72] Constitutive or acquired disorders of complement might also promote PCC independently of the above pathways to further explain the rarity of PCC. Complement disorders have also been described in MIS-C (in older children) and KD and were responsive to IVIG infusion [73, 74]. M-ficolin, an activator of the lectin complement system, was a cluster-defining marker of monocytes detected in bronchoalveolar lavage of COVID-19 patients; soluble M-ficolin was decreased by IVIG treatment of KD [64, 65]. Future studies should include complement in addition to RAS, vasculopathy and fibrosis biomarkers.

Our study, early in the pandemic, offers many new observations that merit further investigation. [1] We confirm ACE2 in blood cells as a biomarker for severe COVID-19-19 (OR 11.8) [2]. ACE2 deficiency may be constitutive in 23% of healthy populations at risk for COVID-19 and PCC. If the absence of ACE2 expression in blood cells is confirmed to be premorbid and constitutive, then efforts at outreach, screening, and prevention of COVID-19 or PCC can target this quarter of the population with high efficiency. Similarly, the design and study of future therapies can be approached with greater economy and ethical benefit. [3] ACE2 deficiency is common over age 70 years. We cannot comment on ACE2 expression in monocytes in children, who have substantially less serious COVID-19 but do develop MIS-C. [4] ACE2 deficiency may respond to plasma products [5]. ACE2 deficiency in monocytes appears independent of other pro-fibrotic and vasculopathic biomarkers that may predispose to PCC.

Our study was performed at a single academic center, with limited sample size and potential for confounding. Assays replicated those published in the literature and were developed in house. The study did not include an ambulatory COVID-19 group because SARS-CoV-2 test availability was limited to inpatients at the time. We cannot discern whether undetectable ACE2 RNA expression in blood cells was constitutive, characteristic of SARS-CoV-2 infection *per se* or of severe disease. We could not study critically ill patients for administrative reasons. Children were not studied. Only a single timepoint was taken, but the study is bolstered by other recent studies published during review [67–69]. Natural immunity and COVID-19 vaccines have modified pathogenesis of COVID-19 and confound analyses of the potential effects of the evolving SARS-CoV-2 virus on ACE2 expression and fibrotic and inflammatory biomarkers. Our assumption of genetic differences in risk of PCC should remain constant despite changes in the pandemic, so this work may inform our understanding of fibrotic diseases or vasculopathies such as Kawasaki disease. Our study should be expanded to include ambulatory SARS-CoV-2 infections as well as PCC and Kawasaki disease.

## Conclusions

We identified ACE2 expression in blood cells as a potential biomarker for severe COVID-19-19 (OR 11.8). Reduced ACE2 expression may be constitutive. The biomarker is rapidly responsive to treatment with convalescent plasma. Untreated, the change in ACE2 persists for weeks during convalescence. TIMP-1, PIIINP, MMP-9 and potentially complement are mutually independent biomarkers that, taken together, may indicate individual, subclinical, pro-inflammatory and pro-fibrotic tone promoting PCC.

## Data Availability

The datasets used and/or analyzed during the current study are available from the corresponding author on reasonable request.
